# Early trajectories and moderators of autistic language profiles: A longitudinal study in preschoolers

**DOI:** 10.1177/13623613241253015

**Published:** 2024-05-21

**Authors:** Kenza Latrèche, Michel Godel, Martina Franchini, Fiona Journal, Nada Kojovic, Marie Schaer

**Affiliations:** 1 University of Geneva, Switzerland; 2 Fondation Pôle Autisme, Geneva, Switzerland

**Keywords:** autism spectrum disorders, cluster analysis, early intervention, Early Start Denver Model, moderators, precision medicine, prognosis

## Abstract

Both expressive and receptive language difficulties in autism emerge early and significantly have an impact on social functioning and quality of life. Despite their wide heterogeneity, autistic language abilities can be stratified into three distinct profiles. Language unimpaired exhibit near-typical verbal performance, language impaired show significant alterations in language structure, and minimally verbal display limited verbal abilities. However, little is known about the early trajectories and moderators specific to each profile. Here, we used a longitudinal sample of 286 preschoolers with autism and 85 typically developing (aged 1.5–5.7 years old), yielding 1164 visits. Our cluster analysis replicated the three well-established autistic language profiles (language unimpaired, *n* = 86; language impaired, *n* = 85; minimally verbal, *n* = 44) and revealed their specific early trajectories in expressive vocabulary, syntax, and pragmatics. At age 2.4, non-verbal cognition moderated the participants’ attribution to each language profile. Moreover, early stereotyped language specifically moderated the later verbal performance of language unimpaired participants, while the language outcome of language impaired participants was moderated by early intensive behavioral intervention. In conclusion, we provided a fine-grained description of language acquisition trajectories and moderators of autistic language profiles in a longitudinal sample exposed to French language, paving the way toward personalized medicine to autistic language difficulties.

**Lay Abstract:**

Language development can greatly vary among autistic children. Children who struggle with language acquisition often face many challenges and experience lower quality of life. However, little is known about the early language trajectories of autistic preschoolers and their moderators. Autistic language can be stratified into three profiles. Language unimpaired experience little to no language difficulties; language impaired show significant difficulties in language; minimally verbal never develop functional language. In this study, we used a longitudinal sample of preschoolers with autism and with typical development (aged 1.5–5.7 years). We replicated the three language profiles through a data-driven approach. We also found that different factors modulated the language outcome within each group. For instance, non-verbal cognition at age 2.4 moderated the participants’ attribution to each language profile. Moreover, early intervention moderated verbal outcome in the language impaired profile. In conclusion, we provided a detailed description of how autistic preschoolers acquire language, and what factors might influence their trajectories. Our findings could inspire more personalized intervention for early autistic language difficulties.

## Introduction

Autism spectrum disorder (ASD) is a set of heterogeneous pervasive neurodevelopmental disorders that is characterized by difficulties in social interactions and communication, accompanied by repetitive behavior and restricted interests (American Psychiatric Association, 2013). In the United States, it is estimated that 1 in every 36 children receives a diagnosis of ASD (Maenner et al., 2023). ASD constitutes a major current challenge for public health politics given its association with a significantly decreased quality of life (QoL) across lifespan (van Heijst & Geurts, 2015). One cause of this reduced QoL lies in the frequent co-occurrence of language impairments in ASD (Tager-Flusberg, 2006). When impaired, the language deficit usually homogeneously affects both its expressive and receptive dimensions (Kwok et al., 2015)—although some levels of expressive/receptive gap have been found in very specific ASD subgroups (Chen et al., 2023). The even expressive/receptive impairment suggests that autistic verbal difficulties are mainly related to a linguistic competence issue, in contrast to an isolated performance disability, according to the traditional cognitivist framework (Chomsky, 1965; Smolensky, 1996). The invaluable advantages conferred by language are illustrated by the famous “*Jack & Jill*” story of the linguist Leonard Bloomfield (Bloomfield, 1984). Bloomfield posits that a speaker can modify another human being’s behavior through language with a level of efficiency and subtlety that could not be achieved by other means of communication. In accordance with Bloomfield’s claim, autistic individuals with lower expressive (EL) and receptive language (RL) abilities lack a crucial communication tool, resulting in more frequent externalizing behaviors such as self-aggression and tantrums (Chan et al., 2023), decreased social functioning (Chow et al., 2022; Miranda et al., 2023), and increased rejection by peers (van der Wilt et al., 2019). Although many types of early interventions can greatly improve verbal outcome in ASD (Brignell et al., 2018; Fuller & Kaiser, 2020; Sandbank et al., 2020), most intervention guidelines remain one-size-fits-all (Fuentes et al., 2021). One reason lies in a current lack of knowledge about which type of intervention will be the most effective for which autistic language profile (Vivanti et al., 2014). One way of achieving this personalized medicine goal (Ozomaro et al., 2013) would rely in the use of deep language phenotyping to achieve a more accurate stratification of the ASD language heterogeneity (Chenausky & Tager-Flusberg, 2022; Robinson, 2012). The aim of deep language phenotyping is to obtain the most precise and comprehensive phenotypic description of individuals’ verbal productions within a given disorder, to eventually help the development of personalized medical approaches (Robinson, 2012). Deep language phenotyping is usually obtained through a combination of targeted direct-speech assessments (Mei et al., 2018). In ASD for instance, deep phenotyping of early verbal trajectories might eventually inspire more targeted intervention guidelines and prognostic estimation in the language domain (Dawson & Sapiro, 2019).

Language abilities across the autism spectrum are very heterogeneous between individuals (Gernsbacher et al., 2016). Nevertheless, there is an emerging consensus that three distinct language profiles exist within ASD (Boucher, 2012; Schaeffer et al., 2023), which have been recently included in the 11th International Classification of Diseases (ICD-11; Harrison et al., 2021; World Health Organization [WHO], 2022). The first canonical language profile is called minimally verbal (MV) and concerns ~20% of autistic individuals (Anderson et al., 2007; Bal et al., 2016; Norrelgen et al., 2015). The MV profile is defined by severe alterations of all linguistic domains, resulting in a very limited vocabulary and minimal syntactic abilities that usually never reach three-word spoken phrases (Norrelgen et al., 2015). Among the approximately 80% of autistic individuals who are not MV, about one half exhibits typical structural language abilities, while the other half experiences significant impairments (Kjelgaard & Tager-Flusberg, 2001; Loucas et al., 2008). Since Tager-Flusberg (2006), those two profiles have been referred to as language normal and language impaired (LI), respectively (Boucher, 2012; Schaeffer et al., 2023). However, we prefer employing the terms language unimpaired (LU) instead to avoid conveying the ableist misconception of ASD (Bottema-Beutel et al., 2021; Canguilhem, 2013). LI individuals have mastered the use of three-word spoken phrases and have developed a substantial vocabulary. They nonetheless exhibit persistent structural alterations that can affect phonology, morpho-syntax, and/or semantics (Boucher, 2012). The degree and type of impairment are very heterogeneous across the LI profile (Kjelgaard & Tager-Flusberg, 2001; Modyanova et al., 2017). In contrast, LU individuals’ verbal intelligence quotient (IQ) and structural language lie within the range of typically developing individuals (TD). Nonetheless, the language of LU individuals often differs from TD in the domains of prosody and pragmatics (Fusaroli et al., 2017; Volden et al., 2009), which might constitute universal linguistic findings in ASD (Boucher, 2012). Moreover, even if the structural language of LU individuals might appear typical in surface, some subtle atypical processes (e.g. mastering of clitics and sentence repetition) have been revealed by linguistic fine-grained explorations (Meir & Novogrodsky, 2020; Terzi et al., 2014) and neuroimaging studies (Harris et al., 2006; Lo et al., 2013; McCleery et al., 2010). Yet, little is known about how early and through which mechanisms these three profiles emerge (Tager-Flusberg, 2016). Language acquisition is an incremental process that relies on innate faculties (Hauser et al., 2002), interaction with environment (Kuhl, 2007) and developmental cascades involving many areas (Karmiloff-Smith, 2018). Consequently, understanding the emergence of distinct language profiles in ASD might only arise from longitudinal deep phenotyping of both linguistic and extra-linguistic development (Karmiloff-Smith, 1998).

Language delay appears as one of the most prevalent early autistic features. It is also the one that leads caregivers to most frequently seek help (Becerra-Culqui et al., 2018; Hudry et al., 2010; Kozlowski et al., 2011). However, studies of language trajectories in autistic preschoolers remain scarce, and it is not clear when canonical profiles become distinguishable. For instance, Tek et al. (2014) identified two distinct language profiles in 17 autistic preschoolers: High-Verbal and Low-Verbal subgroups. In contrast, Smith et al. (2007) identified four distinct trajectories in word production, without any differences in extra-linguistic measures (*n* = 35 autistic preschoolers). By following 192 autistic children between ages 2 and 19, Pickles et al. (2014) found several language trajectories that became stable mostly by the school-age period. The authors of this study pointed toward the importance of examining language trajectories during the preschool period since environment (e.g. early intervention) and brain plasticity might greatly impact language development (Iverson et al., 2023; Werker & Hensch, 2015). Although the reviewed studies brought invaluable understanding of early autistic language trajectories, their sample sizes (except for Pickles et al.) greatly limit the identification of subtle clusters using data-driven approaches (Dalmaijer et al., 2022). Another limitation concerns the lack of deep language phenotyping as all reviewed preschooler studies used aggregate and/or indirect behavioral measures of language (e.g. a measure of communication adaptive functioning in Pickles et al. (Sparrow et al., 2005)). Deeper language phenotyping should involve more fine-grained measures of verbal expressive abilities that cover many linguistic dimensions (Chenausky & Tager-Flusberg, 2022). A valuable contribution to this goal might be brought by parent-reported questionnaires that quantify the child’s productive linguistic abilities (vocabulary inventory, syntax acquisition, pragmatic use). Those tools, like the *Questionnaire sur le Développement du Langage de Production en Français* (DLPF; Bassano et al., 2005), are very time-efficient compared to direct speech assessments by a specialist, while providing excellent concurrent validity in verbal preschoolers (Feldman et al., 2005; Rescorla & Alley, 2001; Ring & Fenson, 2000; Wetherby et al., 2002). Such methods might provide an extensive language phenotyping that lies somewhere between aggregate verbal measures and deep language phenotyping. The current situation is part of a wider preschooler gap in the field of early ASD language research (Ellis Weismer et al., 2010; Godel et al., 2023; Hudry et al., 2010; Tek et al., 2014; Yankowitz et al., 2019).

In this study, we aim to address the “preschooler gap” in terms of language profiles by combining data-driven and extensive language phenotyping approaches in a large longitudinal cohort of preschoolers with either TD or ASD. We explored the distinct early language acquisition trajectories within each ASD language profile in a longitudinal sample of 371 preschoolers (286 with ASD and 85 with TD) aged from 1.5 to 5.7 years old (y.o.). Verbal developmental quotients (DQs) were used as an aggregate measure of language outcome while longitudinal extensive language phenotyping was achieved using the DLPF. We explored the moderators of verbal outcome using demographic and early non-verbal behavioral measures. We had three main aims and hypotheses. First, we expected to find the three canonical language groups (MV, LI, and LU) within our autistic preschoolers using a data-driven clustering approach. To our knowledge, this assumption has never been tested on this age group thus far. Second, we aimed at providing a longitudinal extensive language phenotyping (including vocabulary, grammar, and pragmatic expressive abilities) for each cluster. We expected that each profile would follow its own specific early linguistic trajectories, providing some fine-grained developmental norms of language development. Third, we explored the early behavioral, environmental (early intervention), and demographic measures that could have moderated the participants’ language profile attribution. We also tried to find moderators that affected the individuals’ verbal outcome within each profile.

## Methods

### Participants

Our sample was part of an ongoing open longitudinal cohort—the Geneva Autism Cohort (Franchini et al., 2018). The cohort follows preschoolers with either ASD or TD and collects longitudinal child behavioral measures (see “Measures” subsection for a detailed description). Since 2012, preschoolers have been recruited through announcements in the Geneva community (e.g. parent associations and clinical centers). For autistic participants, diagnosis was set at the initial time point by the licensed child psychiatrist MS using the *Diagnostic and Statistical Manual of Mental Disorders* (5th ed., *DSM*-V) diagnostic criteria. Diagnosis was further confirmed by the diagnostic cutoffs of the Autism Diagnostic Observation Schedule (ADOS). Prior to inclusion, all TD participants were screened for the absence of any developmental concern, for the absence of any neurological and somatic concerns that might have affected their development, and for the absence of any ASD diagnosis in their first-degree relatives. All TD participants underwent an ADOS to exclude the presence of an ASD diagnosis using the ADOS diagnosis cut-offs (highest calibrated severity score in the TD sample was 3), and we made sure that none of the TD participants showed any significant developmental delay as measured with either the Mullen Scale of Early Learning (MSEL) or the Psychoeducational Profile—third edition (PEP-3) using a cut-off of 70 in the composite DQ (lowest composite DQ in the TD sample was 84). Written informed consent forms were signed and provided by the participants’ caregivers. The Ethics Committee of the University of Geneva approved the research protocol. The open cohort longitudinal design involves assessments conducted every 6 months over 2 years (hence totalling five time points per participant when the follow-up is completed). Our final sample comprised 371 participants (1164 time points) aged from 1.5 to 5.7 y.o. The TD group comprised 85 participants (221 time points, age range 1.5–5.6 y.o., 44.7% of female biological sex) and the ASD group 286 (943 time points, age range 1.5–5.7 y.o., 17.5% of female biological sex). Sample characteristics are detailed in Table 1. Moreover, Supplementary Figure S1 provides an illustration of the recruitment process with participant’s age at each visit. For any longitudinal time point to be included, the participant had to be younger than 68 months old. This age corresponds to the upper limit of the MSEL that was used to compute the DQs (see the “Measures” subsection). Participants that were not exposed to the French language were not included (i.e. not part of the *n* = 371 final sample) to get a homogeneous sample in terms of language exposition (48 participants, 8 TD and 40 ASD). There was no community involvement in the reported study.

**Table 1.** Sample characteristics with statistical comparison between TD and ASD.

Measure (mean (*SD*))	TD	ASD	*p*-value
Number of participants	85	286	
Number of time points	221	943	
Time points per participant	2.6 (1.2)	3.3 (1.5)	< **0.001**
Mean age (years)	3.4 (0.9)	3.6 (0.8)	0.088
Age range (years)	1.5–5.6	1.5–5.7	
Female biological sex	38 (44.7%)	50 (17.5%)	< **0.001 (**χ^2^ **)**
Plurilingual environment	28 (32.9%)	145 (50.7%)	** 0.004 (**χ^2^ **)**
College degree completed	73 (91.7%) (*n* = 84)	151 (56.1%) (*n* = 269)	< **0.001 (**χ^2^ **)**
Mean ADOS CSS total	1.1 (0.2)	7.4 (1.6)	< **0.001 (MW)**
Mean ADOS CSS SA	1.1 (0.3)	6.4 (1.6)	< **0.001 (MW)**
Mean ADOS CSS RRB	2.3 (1.8)	9.0 (1.3)	< **0.001 (MW)**
Mean composite DQ	112.8 (10.8)	70.2 (23.5)	< **0.001**
Mean expressive language DQ	105.0 (15.3)	58.0 (25.4)	< **0.001**
Mean receptive language DQ	117.5(12.4)	62.9 (30.1)	< **0.0001**
Mean visual reception DQ	122.3 (16.3)	82.7 (25.6)	< **0.001**
Mean fine motor DQ	106.1 (13.0)	77.1 (19.3)	< **0.001**
Mean PSI stress total	59.1 (18.0) (*n* = 79)	90.5 (22.8) (*n* = 241)	< **0.001**

TD: typically developing individuals; ASD: autism spectrum disorder; ADOS: Autism Diagnostic Observation Schedule; CSS: calibrated severity score; SA: social affect; RRB: restricted, repetitive behavior; DQ: developmental quotient; PSI: parental stress index.

For categorical variable, chi-square was applied (χ^2^). For continuous variables, we used either two-tailed independent *t*-tests or Mann–Whitney test (MW) when normality was not assumed (e.g. with ADOS CSS). The *p*-values < 0.05 are highlighted in bold. For variables that change over time (e.g. DQ), we computed mean, *SD*, and *t*-statistics using each participant’s averaged value over time points.

### Measures

We collected three types of measures. First were measures of language outcome (verbal DQs), used to define language profiles. Second were descriptive measures, in the form of longitudinal extended language phenotyping of expressive vocabulary, grammar, and pragmatics with the *Questionnaire sur le Développement du Langage de Production en Français* (DLPF). As a secondary descriptive measure, we also collected the Parental Stress Index Short Form (PSI-SF). Third were measures of potential moderators of language outcome (demographic measures, non-verbal cognition, and autistic symptoms).

#### Outcome measure: verbal DQ

To measure verbal performances, we used the Mullen Scales of Early Learning (MSEL; Mullen, 1995), which is a standardized tool assessing children aged 0 to 68 months. The MSEL offers a time-efficient, comprehensive assessment of cognitive, motor, and verbal domains that maximizes participant engagement and may reduce potential fatigue effects that are common when assessing young children with autism or typical development. We computed distinct DQ scores for each language scale, namely RL and EL. Each DQ score was obtained by dividing the developmental age (i.e. age equivalent score of a scale) by the child’s chronological age and multiplying by 100 (Lord et al., 2006; Shen et al., 2013). Unlike standard scores, DQ provides reliable and age-normalized metrics while mitigating floor-effects of very low performing participants.

The MSEL was not administered to a small proportion of our total sample (102 time points, 8.8%) because it was added later to the research protocol. For missing MSEL data, we used substitute DQ measures in EL and RL obtained with the PEP-3 (Schopler et al., 2005). The PEP-3 is another standardized developmental evaluation designed for children aged from 2 to 7 years. As we did for the MSEL, we computed DQs for EL and RL domains. To support the interchangeability of the MSEL and PEP-3 language DQs, we selected the 467 time points (278 participants) for which both evaluations were administered. Excellent consistency across the tools was found for both EL (Cronbach’s alpha = 0.899) and RL (Cronbach’s alpha = 0.913) in our sample.

#### Primary descriptive measure: the *Développement du langage de production en français* for longitudinal extended language phenotyping

The *Développement du langage de production en français* (DLPF) is a standardized parent-reported questionnaire that aims at assessing the development of EL in children exposed to French language aged from 18 to 42 months (Bassano et al., 2005). There are four versions of the DLPF depending on the child’s age. In all versions of the DLPF, the questionnaire is divided into three linguistic sections exploring lexical, grammatical, and pragmatic development, respectively. We rated all responses following the authors’ scoring guide (Bassano et al., 2020), leading to three separate scores.

In the lexical section, parents are presented lists of words and asked to check off all the words their child can produce. The total vocabulary estimates the child’s vocabulary size in number of words. Then, the grammatical section investigates grammatical forms (e.g. the use of articles, noun plurals or verbal tenses), as well as structures and complexity of word combinations. Parents completing the grammatical section are either asked to evaluate the frequency (i.e. Never, Sometimes, or Often) with which their child produces a form, or to indicate whether their child uses a specific formulation. By scoring the responses (Never were recoded as 0, whereas Sometimes and Often were recoded as 1), we obtained a total grammatical score. Finally, in the pragmatic section, parents are asked to evaluate the frequency of their child’s participation in conversation, language use in various contexts, and the organization of sentences for more complex communication (i.e. Never, Sometimes, or Often). The responses were recoded as previously described (Never as 0, Sometimes and Often as 1), yielding a Total Pragmatic Score.

The total vocabulary score is transparent, that is, it represents the estimated raw number of words expressed by a participant. In contrast, the total grammatical score is quite opaque. However, one of its items, the two-word combination acquisition, represents a clinically relevant and transparent measure. A child’s ability to combine two words is considered as marking the emergence of his.her productive syntax (Braine & Bowerman, 1976), and it is a crucial syntactic milestone used to stage verbal abilities in ASD, for example, in both the ADOS and the revised Autism Diagnosis Interview (Lord et al., 1994, 2012). Consequently, we included the DLPF two-word combination acquisition item as a supplementary language descriptive measure, coded as a binary categorical variable (acquired or not acquired).

In TD, the study of Bassano et al. (2005) showed a plateau effect when approaching 42 months, thus limiting the DLPF clinical significance in TD after this age. Nonetheless, we administered the DLPF to children up to 68 months, because we expected a later plateau in children with ASD given their frequent language delay.

#### Secondary descriptive measure: parental stress index

To describe the impact of language impairment (language profile and language outcome) on the parental QoL, we used the parenting stress index—short form (PSI-SF, Abidin, 1995). Parental stress has been shown to be an important mediator of QoL (Wang et al., 2022). We used the total stress score (which is the sum of three subscales) as our measure of parental QoL.

#### Outcome moderator measure 1: parental socio-economic status

We measured the participant’s social-economic status with the highest level of education achieved by parents (Hollingshead, 1975). Parental education level was categorized as either (1) elementary school or high school completed or (2) college degree completed. Parental education level is commonly used as a reliable proxy for parental socio-economic status in studies exploring early language development (Bergelson et al., 2023; Bornstein, 2003; Hoff, 2003). In TD, parental socioeconomic status has been reported to affect the child language acquisition, for example, through differences in parent–child interaction and/or availability of learning resources (Pace et al., 2017)

#### Outcome moderator measure 2: bilingual exposure

Plurilingual environments can affect the rate of lexical acquisition in TD (Bialystok & Craik, 2010), even though a bilingual environment has not been associated with ASD verbal outcome (Drysdale et al., 2015; Hambly & Fombonne, 2012). In our study, a monolingual environment was defined by an exclusive exposure to French, and a plurilingual environment by an exposure to French and at least one other language.

#### Outcome moderator measure 3: biological sex

The biological sex of the participants has also been explored as a possible moderator of language outcome since early verbal differences between boys and girls have been reported in both TD (Oller et al., 2020) and autistic samples (Carter et al., 2007; Chenausky et al., 2018).

#### Outcome moderator measures 4: early non-verbal cognition and fine motor skills

To measure non-verbal cognition before the age of 3, we used the visual reception (VR) and for assessing fine motor (FM) skills, we used FM domains of the MSEL. We computed separate DQ scores for both domains. When the MSEL was not available, we used the corresponding domains in the PEP-3, namely FM and verbal–preverbal cognition (CVP). Early motor skills have been associated with later RL skills in children with ASD (Hannant, 2018). In addition, visuospatial cognition has been associated with later RL and EL skills in preschoolers with ASD (Hellendoorn et al., 2015). Some studies also showed that FM skills were associated with language outcomes in siblings at high familial likelihood of autism (Hwang & Lee, 2022; LeBarton & Iverson, 2013).

#### Outcome moderator measures 5: early autistic symptoms

The ADOS, second edition (ADOS-2; Lord et al., 2012) comprises several semi-structured activities that quantify symptoms in two domains: social affect (SA) and restricted and repetitive behaviors or interests (RRB). The ADOS-2 comprises five modules that depend on the child’s age and language level. To compare scores across modules we used calibrated severity scores (Gotham et al., 2009; Hus et al., 2014) to obtain SA and RRB severity scores. The ADOS-2 were administered by trained examiners and video recorded for coding. Early ASD symptom severity has been associated with language outcome (Loucas et al., 2008; Thurm et al., 2015).

#### Outcome moderator measures 6: early intervention program

Among the 286 autistic participants, 98 (34.3%) underwent an individualized 2-year early intensive intervention program following the Early Start Denver Model (ESDM; Rogers and Dawson, 2010). As a Naturalistic Developmental Behavioral Interventions (NDBIs; Schreibman et al., 2015), the ESDM program integrates principles from developmental science and behavioral learning, such as emphasizing the importance of developmental prerequisites and promoting child engagement in social interaction using motivating activities. Studies have recognized the ESDM as an effective intervention that significantly increases cognitive, verbal, and adaptive skills (Dawson et al., 2010; Fuller et al., 2020; Fuller & Kaiser, 2020). Participants enrolled in the ESDM intervention program received between 15 and 20 h a week of individual sessions with a graduate-level therapist trained with the ESDM approach. Children underwent evaluation every 3 months throughout the 2-year intervention period and their parents also received coaching sessions at the beginning of their child’s enrollment in the program. For more details regarding the ESDM program in Geneva, see the study by Godel et al. (2022).

### Statistical analyses

#### Identifying the outcome time point: preliminary analysis

Since participants have many longitudinal verbal assessments, we had to decide which time point best reflected verbal outcome (verbal DQ), to then input in the cluster analysis. We had two criteria for determining outcome time point. First, we wanted to determine the age by which verbal DQs were stable, as labile outcome measures could compromise cluster quality. Indeed, when we ran a preliminary analysis to estimate the verbal DQ trajectories in our sample, we found an overall significant quadratic effect of age in the autistic sample, supporting the need to identify the age at which DQs start to remain stable. Then, the selected outcome time points had to be approximately the same age across participants to minimize age heterogeneity. Here are the steps we followed. We first computed the age effect on verbal DQ using a sliding window of 12 months width and 1 month increment (see Supplementary Figure S2). Within each 12-month window (e.g. for the period going from 12 to 23 months of age), a linear mixed-effect model was applied to test the effect of age on language (RL and EL), using all time points collected in the ASD group. Linear model included between-subject fixed effect and random intercept to predict verbal DQ with age (Matlab^®^ R2018b function *fitlme*). False discovery rate (FDR) correction was applied over all the age bins tested.

We found that by 3.75 y.o. months, age had no significant effect anymore on RL (i.e. *p* > 0.05 in all sliding windows from 3.75 y.o. and older). Before this age, age had a positive significant effect on RL DQ in all windows. For EL, stability of DQ (i.e. non-significant age effect) was reached a bit earlier, at the age of 3.50 y.o. This means that after those ages (3.75 and 3.50 y.o., respectively), RL and EL DQs were globally stable in the autistic group. When removing the time points collected before 3.75, the average age of autistic participants was 4.4 y.o. Consequently, we defined the outcome time point (i.e. time point from which verbal DQs were used to cluster) as the participants’ time point nearest to 4.4 y.o.

#### Identifying the language profiles: cluster analysis

We used a data-driven cluster analysis approach to stratify our ASD sample into distinct language profiles based on participants’ verbal outcome. We applied the SPSS^®^ in-built TwoStep clustering algorithm (SPSS^®^ IBM^®^ Statistics 26.0 for macOs (Armonk, NY: IBM Corp.) using the default parameters suggested by the SPSS^®^ manual (Chiu et al., 2001; Norušis, 2006). One advantage of this clustering approach is that it automatically determines the optimal number of clusters, without any a priori. Briefly, the standard TwoStep cluster analysis procedure sequentially tests 15 clustering solutions (incrementally ranging from 1 to 15 clusters). For each cluster solution, we used two input variables: EL and RL DQs at outcome. The Akaike’s information criterion (AIC) is computed for each clustering solution. The optimal solution is then automatically determined by SPSS^®^ based on a compromise between the largest ratio of AIC change and the largest ratio of distance measures. The ratio of AIC changes is defined as the AIC changes between an *n* cluster solution and an *n* *+* 1 cluster solution, normalized on the AIC change between the one- and the two-cluster solutions. The ratio of distance measures of a given *n* cluster solution is computed relatively to the *n*–1 cluster solution, using a log-likelihood measure of the input variables (i.e. likelihood to observe the input variables in a given cluster solution). Once the optimal cluster solution has been automatically determined, its overall quality is estimated by the silhouette coefficient of coherence and separation, which ranges from −1 to 1. In addition, the normalized importance score (ranging between 0 and 1) estimates the relative contribution of each input variable to the optimal cluster solution. Once the language profiles were identified, we provided a description of their characteristics at outcome (see Supplementary Table S1). We carried out an analysis of variance (ANOVA) test (or chi-square for categorical variables) on the parental and child measures collected at outcome time point (4.4 y.o.) with two-by-two post hoc comparisons with Bonferroni correction.

#### Describing each profile’s extensive language phenotypes: longitudinal analyses

We used a mixed modeling method to investigate the extensive language phenotype trajectories (using DLPF measures: total vocabulary, total grammar score, total pragmatic score, and the additional two-word combination acquisition) over time within each ASD language profile. Mixed modeling has been successfully used to measure longitudinal changes in cognition in developmental disorders including ASD (Latrèche et al., 2021; Maeder et al., 2016). Age and group (language profile) were modeled as fixed effects while DLPF measures as random effects. Random slope model analysis was carried out using the *my Mixed Model Trajectories* toolbox (available publicly https://github.com/danizoeller/myMixedModelsTrajectories) implemented in MATLAB^®^ R2019b (MathWorks). We estimated language trajectories between the profiles by fitting random-slope models (constant, linear, quadratic), each corresponding to a specific relationship between age and one language measure (DLPF Total Vocabulary, DLPF Grammar Score, DLPF Pragmatic Score). We used the Bayesian information criterion that enabled us to select the most suitable model order. In our case, the quadratic model order was consistently selected for all the trajectories. For any statistically significant effect across the language profiles, we used a one-to-one profile post hoc comparison applying Bonferroni correction. For the longitudinal trajectories of the two-word combination acquisition (categorical variable), we used a chi-square test with an age sliding window of 6 months (corresponding to the average time-lapse between time points) with an increment of 1 month. We compared the proportion of participants who had acquired two-word combinations at each age frame across the ASD language profiles. FDR correction was applied over all the tested age bins.

#### Moderators of verbal outcome

First, we examined whether early factors could have led some participants to express one language profile instead of the others. We used ANOVA or chi-square across the profiles using all the eight outcome moderator measures. Then, we tested whether those moderators might have specifically affected the language outcome within each language profile. Given the relatively large number of moderators to test (eight), we decided to use a single outcome variable by averaging the EL and RL DQs. This resulted in one outcome Verbal DQ for each participant, which summarizes his.her verbal abilities at age 4.4. This approach allowed us to downsize the number of analyses and is supported by the absence of any significant expressive/receptive developmental gap within our ASD sample.

Within each language profile, we applied one linear regression model to test the effect of continuous moderators (DQs and ADOS values at age 2.4) on the verbal outcome. For categorical moderators (biological sex, parental education, bilingual exposure, and early intervention), we applied a two-tailed *t*-test to test if the variable was moderating the Verbal Outcome. For each moderator, the *p*-value was Bonferroni corrected for the number of language profiles tested. The statistical significance threshold for Bonferroni corrected *p*-values was set at alpha = 0.05. ANOVA, chi-square, *t*-tests, and linear regressions were performed on IBM^®^ SPSS^®^ Statistics 26.0 for macOs (Armonk, NY: IBM Corp.).

## Results

### Characteristics of TD and ASD samples

The ASD (*n* = 286, 943 time points) and TD (*n* = 85, 221 time points) groups showed no difference in age across all time points (mean age 3.6 ± 0.8 for ASD and 3.4 ± 0.9 y.o. for TD). There were significantly more TD females (44.7%) than ASD (17.5%). Parental education was significantly higher in the TD group. As expected, the ASD group had higher autism symptom severity (7.4 ± 1.6 ADOS CSS compared to 1.1 ± 0.2 in TD). The ASD group also exhibited lower developmental skills (70.2 ± 23.5 composite DQ compared to 112.8 ± 10.8 in TD). Regarding parental stress, parents of children with ASD self-reported significantly higher stress (90.5 ± 22.8 in PSI-SF total stress compared to 59.1 ± 18.0 in TD). Behavioral and demographic differences between ASD and TD are summarized in Table 1.

### Identification of ASD language profiles using cluster analysis

The TwoStep cluster analysis automatically determined that the three-cluster solution was the optimal one (ratio of AIC changes = 0.375, ratio of distance measures = 3.658, results for all cluster solutions provided in Supplementary Material 1). This optimal three-cluster solution showed a silhouette measure of coherence and separation equal to 0.6, which corresponds to a “good” cluster quality (Norušis, 2006). Both EL and RL input variables contributed equally to this three-cluster solution, with an importance score of 1.0 for both EL and RL. The description of clusters’ characteristics at outcome are reported on Supplementary Table S1, and longitudinal descriptions of the clusters are illustrated in Supplementary Material.

### Language profiles characteristics

The first cluster consisted of the 20.5% ASD participants that were MV at age 4.4. At this age, their verbal performance was more than 4 standard deviations below that of TD participants (21.6 ± 6.9 EL DQ and 21.7 ± 10.6 RL DQ).

Then, the second cluster was made of 40.0% of the autistic participants who presented a significant language impairment at outcome. Their verbal performance fell within a range of 2 to 3 standard deviations below TD at age 4.4 (55.2 ± 13.8 in EL DQ and 62.1 ± 14.3 in RL DQ), indicating a less significant delay compared to MV individuals at the same age.

The third cluster comprised 40.0% of autistic participants whose language was unimpaired (LU) at outcome. Their verbal outcome fell within the range of TD at 4.4 y.o. (90.0 ± 11.5 EL DQ and 101.5 ± 14.1 RL DQ) with vocabulary, grammar, and pragmatic scores significantly above the ones of LI and MV individuals.

**Figure 1.**
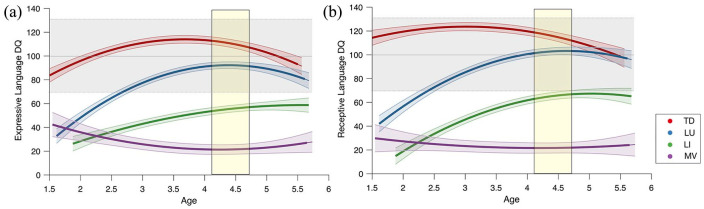
Expressive and receptive developmental trajectories in the three ASD language profiles. (a) Expressive Language DQ. (b) Receptive Language DQ. TD (in red) were not included in the statistical comparison and their trajectory is only displayed for illustration purposes. The colored bands around the estimated group-level trajectory indicate the 95% confidence interval. Yellow boxes highlight the DQs at outcome, that is, the variables used as input for the Cluster Analysis. DQ: developmental quotient; LI: language impaired; LU: language unimpaired; MV: minimally verbal; TD: typical development.

We provide an illustration of the three clusters’ trajectories in terms of EL and RL DQs on Figure 1. The yellow boxes correspond to the DQs at age 4.4 ± 0.3, that is, the outcome values used as input into the TwoStep cluster analysis to define the language profiles. Each cluster differed from the others in terms of group and group × age effect for both EL and RL trajectories (see Supplementary Table S2).

Regarding other measures at outcome, LI and LU individuals did not differ in autistic symptoms (7.2 ± 1.8 and 6.8 ± 1.7 total ADOS CSS, respectively) while MV individuals showed more autistic symptoms (8.6 ± 1.3 total ADOS CSS) compared to the two other clusters. This difference was driven by symptoms in the communication and social interaction domains. We also observed a decreasing gradient from LU to MV profiles in FM and VR performances at outcome. Finally, parental QoL did not differ between clusters, with all three language profiles showing similar amounts of parental stress (PSI-SF total stress) at the age of 4.4 years (87.2 ± 26.6 in LU; 91.4 ± 30.6 in LI; 101.1 ± 19.3 in MV; *p* = 0.104).

### Longitudinal extensive language phenotype of each language profile

**Figure 2.**
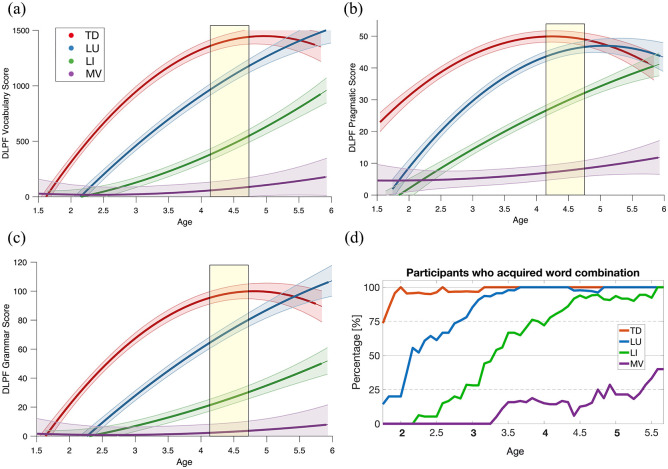
Longitudinal extensive language phenotyping of the three ASD language profiles. (a) DLPF vocabulary score, (b) DLPF pragmatic score, (c) DLPF grammar score, (d) DLPF proportion of word combination. TD (in red) were not included in the statistical comparison and their trajectory is only displayed for illustration purposes. The colored bands around the estimated group-level trajectory indicate the 95% confidence interval. Yellow boxes highlight the age window at which Language DQ variables were sampled to define the clusters. DLPF: *questionnaire du développement du langage productif en français*; LI: language impaired; LU: language unimpaired; MV: minimally verbal; TD: typical development.

Then, we explored the early language trajectories in the domains of vocabulary, grammar, and pragmatics using the DLPF measures (see Figure 2, Supplementary Table S2). The three autism profiles (LU, LI, MV) showed different verbal trajectories on all linguistic metrics (see Figures 1 and 2). We found significant differences in group effect (*p* *<* 0.001) and in group × age effect (*p* *<* 0.001) across all profiles and in all metrics (see Figures 1 and 2(a) to (c)). Overall, LU individuals demonstrated a delayed onset of linguistic acquisition compared to TD, but with rates close to TD (see Figure 2(a) to (c)). Note that the figures with the individual data are available in the Supplementary Material (see Supplementary Figure S3). By age 2, ~50% of LU children had achieved two-word combinations (compared to ~100% in TD at the same age). LI children exhibited a delayed onset and slower rate of language acquisition compared to both TD and LU children. Approximately 50% of them had acquired two-word combinations by age 3 and almost all of them had reached this milestone by age 4.4 (see Figure 2(d)). Finally, the language acquisition of MV children was characterized by minimal acquisitions in the three linguistic domains, although the pragmatic domain seemed relatively less affected compared to vocabulary and grammar. Shortly before age 3.5, we identified the first MV participant who achieved two-word combinations. By the age of 5, ~25% of them had acquired this milestone. Similar patterns between the three profiles were found regarding the DLPF subscales (see Supplementary Figure S4).

### Moderators of language profile assignation

**Figure 3.**
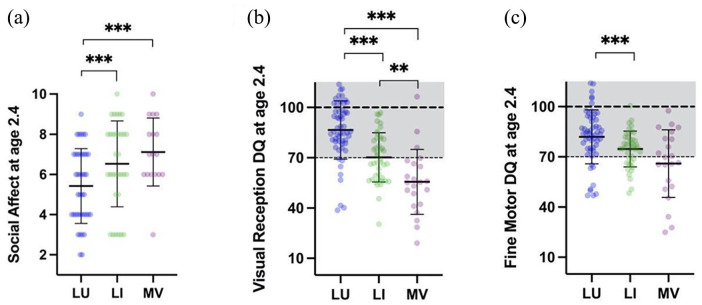
Moderators of language profile attribution. (a) Differences between the three language clusters in early autistic symptoms in the domain of Social Affects (at ~2.4 y.o.) (b) Early differences between clusters in non-verbal developmental measure of visual reception (at ~2.4 y.o.) (c) Early differences between clusters in non-verbal developmental measure of fine motor skills (at ~2.4 y.o.). Solid lines indicate the mean and standard deviation of the data. On panels (b) and (c), the dotted lines and the shaded gray area indicate the expected value for age (100) in typical development, and the threshold commonly used to define a significant delay (70). DQ: developmental quotients; LI: language impaired; LU: language unimpaired; MV: minimally verbal. *p < 0.05; **p < 0.01; ***p < 0.001 after Bonferroni correction for three comparisons.

We conducted ANOVA or chi-square analyses to assess differences between the three language profiles for each of the six moderator variables (“Methods” section). The proportion of participants with female biological sex in the MV profile (25.0%) was higher than in the LU profile (8.1%; see Table 2). Plurilingual exposure and parental education level showed no statistically significant difference between language profiles. The absence of differences in parental education levels also reduces the probability of bias in the DLPF reports (Roberts et al., 1999). In contrast, the participation in an early intervention program (ESDM) moderated the cluster attribution (*p* = 0.042) with a higher proportion of LU participants having received ESDM (54.7% of LU) compared to MV (31.8% of MV). Regarding early behavioral differences between groups (2.4 ± 0.3 y.o.), LU individuals exhibited fewer symptoms in social affect (6.1 ± 2.0 SA) compared to both other profiles (8.0 ± 1.9 in LI and 8.2 ± 1.5 in MV; see Figure 3(a)). In terms of early cognition, each profile showed significantly different VR DQ at age 2.4. LI showed the highest values (86.5 ± 17.4) and MV the lowest (55.6 ± 19.4) (see Figure 3(b)). Early VR was the only moderator that showed statistical difference across all profiles. Early FM skills only differed between LI (92.0 ± 16.1) and MV (66.0 ± 20.2; see Figure 3(c)).

**Table 2.** Moderators of language profile attribution (LU, LI, and MV).

Moderators of cluster attribution	Language unimpaired	Language impaired	Minimally verbal	*p*-value	LU/LI	LU/MV	LI/MV
Female biological sex	7 (8.1%)	11 (12.9%)	11 (25.0%)	**0.028 (**χ^2^ **)**	.918	**.025**	.252
Plurilingual environment	41 (47.7%)	38 (44.7%)	25 (56.8%)	0.421 (χ^2^)			
College degree completed	52 (61.9%) (*n* = 84)	43 (53.1%) (*n* = 81)	24 (55.8%) (*n* = 43)	0.508 (χ^2^)			
I-ESDM (2 years 15–20 h/week)	47 (54.7%)	37 (43.5%)	14 (31.8%)	**0.042**	0.438	**0.042**	0.591
ADOS CSS SA at age 2.4	6.1 (2.0)	8.0 (1.9)	8.2 (1.5)	< **0.001 (KW)**	< **0.001 (MW)**	< **0.001 (MW)**	1.000 (MW)
ADOS CSS RRB at age 2.4	8.1 (1.8)	8.6 (1.8)	9.2 (1.2)	**0.020 (KW)**	0.345 (MW)	**0.018 (MW)**	0.627 (MW)
Visual reception DQ at age 2.4	86.5 (17.4)	74.7 (10.8)	55.6 (19.4)	< **0.001**	< **0.001**	< **0.001**	**0.004**
Fine motor DQ at age 2.4	92.0 (16.1)	74.7 (10.8)	66.0 (20.2)	< **0.001**	0.065	< **0.001**	0.096

For categorical variables, chi-square was applied (χ^2^). For continuous measures we used one-way analysis of variance (ANOVA) with post hoc *t*-tests or Kruskal–Wallis (KW), and post-hoc Mann–Whitney (MW) when normality was not assumed (e.g. for the ADOS CSS). The *p*-values < 0.05 are highlighted in bold. Post-hoc *p*-values are Bonferroni-adjusted for three comparisons.

### Within-profile moderators of verbal outcome

**Figure 4.**
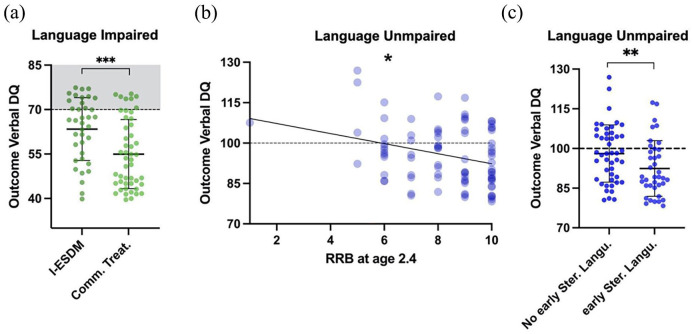
The early factors that specifically moderated the language outcome within each profile. (a) In language impaired (LI), participants who underwent a 2-year individualized early intensive naturalistic behavioral intervention (Early Start Denver Model, ESDM) showed higher verbal outcome at age 4.4. (b) In language unimpaired (LU), RRB at 2.4 y.o. were associated with poorer verbal outcome at age 4.4. (c) Post hoc analyses revealed that stereotyped language was the early RRB feature that mostly drove the effect in panel (b). On panels (a) and (c), solid lines indicate the mean and standard deviation of the data. The dotted lines and the shaded gray area indicate the expected value for age (100) in typical development, and the threshold commonly used to define an important delay (70). I-ESDM: individualized ESDM; RRB: repetitive and restricted behavior. *p < 0.05; **p < 0.01; ***p < 0.001.

We tested within each language profile what factors could moderate the individuals’ verbal outcome at age 4.4 (see Table 3, Figure 4). For categorical variables, a two-tailed *t*-test was run and for continuous variables, a linear regression was applied. We found that participation in a 2-year individual early intensive intervention program (the Early Start Denver Model, ESDM) was associated with higher verbal outcome at age 4.4 in the LI profile (*p* < 0.001, *t*-stat = 3.4, see Figure 4(a), but it did not reach significance threshold in LU and MV profiles (see Table 3). In the LU profile, we found that less RRB symptoms at age 2.4 years was associated with higher verbal performances 2 years later (*p* = 0.048; Estimate = –1.837; *R*^2^ = 0.104, see Figure 4(b)). Statistical significance held even when removing the outlier participant with an early RRB score of 1. Other tested moderators showed no significant association with verbal outcome in any of the language profiles (see Table 3). In MV individuals, no measure in either demographic, intervention, or early behavior significantly predicted the verbal outcome.

**Table 3.** Within-profile moderators of verbal outcome.

Within-cluster moderators of language outcome	*p*-value	LU (*n* = 86)		*p*-value	LI (*n* = 85)		*p*-value	MV (*n* = 44)	
Categorical moderators		*t*-stat			*t*-stat			*t*-stat	
Female biological sex	1.000	0.0		1.000	0.2		0.806	1.1	
Plurilingual Environment	0.555	1.3		0.063	−2.4		0.996	1.0	
College degree completed	1.000 (*n* = 84)	0.0		1.000 (*n* = 81)	−0.5		0.201 (*n* = 43)	1.9	
I-ESDM (20 h/week for 2 years)	1.000	−0.8		< **0.001**	**–3.4**		1.000	−0.44	
Continuous	*p*-value	Estimate	*R* ^2^	*p*-value	Estimate	*R* ^2^	*p*-value	Estimate	*R* ^2^
ADOS CSS SA at age 2.4	1.000 (*n* = 56)	0.228	0.002	0.717 (*n* = 41)	−1.066	0.035	0.372 (*n* = 20)	−1.740	0.114
ADOS CSS RRB at age 2.4	**0.048**	**–1.837**	**0.104**	0.654	−1.232	0.039	1.000	−0.480	0.005
Visual reception DQ at age 2.4	0.126 (*n* = 56)	0.165	0.074	0.879 (*n* = 43)	0.121	0.027	0.450 (*n* = 22)	0.127	0.101
Fine motor DQ at age 2.4	0.276	0.097	0.022	0.399	0.236	0.054	0.348	0.133	0.119

Within each language profile (LU, LI, and MV), we tested the effect of eight moderators on the verbal DQ at outcome (4.4 years old). Verbal DQ is the mean between expressive and receptive DQs. For continuous moderators, linear regressions were used. For categorical moderators, *t*-tests were used. We display the Bonferroni corrected *p*-value (for each moderator, correction for three comparisons). Statistically significant models are highlighted in bold.

Since RRB at age 2.4 showed a significant association with verbal outcome in LU individuals, we ran a post hoc analysis to understand which RRB symptom was driving this effect. We looked at each of the five items of RRB in the ADOS Toddler Module (Luyster et al., 2009), that is, intonation of vocalizations/verbalizations, stereotyped/idiosyncratic use of words or phrases, unusual sensory interests, hand and finger movements/posturing, and unusually repetitive interests or stereotyped behaviors. For each RRB symptom, we compared the verbal outcome of LU participants presenting the symptoms to those who did not. Independent samples two-tailed *t*-tests were used. The *p*-values were Bonferroni corrected for five comparisons (one *t*-test per RRB item). We found that the presence of stereotyped and/or idiosyncratic use of words or phrases at 2.4 y.o. was specifically associated with lower verbal outcome in LU participants (Bonferroni corrected *p* = 0.010, *t*-stat = −3.2, see Figure 4(c)).

## Discussion

In this study, we first aimed at identifying the three canonical autistic language profiles within a large sample of preschoolers using a data-driven cluster analysis. Then, we aimed at providing a fine-grained description of the early language acquisition trajectories within those three ASD language profiles. Finally, we tried to identify some specific features (biological sex, environment, and non-verbal behavioral characteristics at 2.4 y.o.) that might have moderated the participants’ profile attribution and their verbal outcome.

First, our unbiased cluster analysis (TwoStep algorithm) determined that a model with three distinct language profiles at age 4.4 was a better fit than a single ASD group exhibiting a continuum of language difficulties. As the language skills of autistic preschoolers were stable at this age (see Supplementary Figure S2), we were able to classify participants into the three well-established ASD language profiles: LU, LI, and MV (see Supplementary Table S1). Global stability of verbal difficulties around the age of 4–5 y.o. was expected given previous studies on critical windows in language development (Everitt et al., 2013). Although the identification of three ASD language clusters has been reproduced in various studies involving adult and school-aged ASD populations (Boucher, 2012; Schaeffer et al., 2023; Silleresi et al., 2020; Tager-Flusberg, 2006), our study is the first to date that identifies these profiles in a preschooler sample encompassing the full autistic spectrum. The correspondence between our three preschooler profiles and the ones previously identified in older populations is supported by several arguments. First, we found that the verbal DQs in ASD remained stable by the age of 3.8 (see Supplementary Figure S2). Consequently, our participants’ profile attribution will likely remain stable with age given that their verbal DQs are unaffected by age. Furthermore, our early profiles are very similar to the canonical ones regarding their relative prevalence and language structure. Our MV cluster represented 20.5% of our ASD sample. At 4.4 y.o., MV individuals showed scarce vocabulary on average (103 words, Supplementary Table S1) combined with very limited grammar abilities (82.8% of them unable to produce two-word phrases). This corresponds to classical MV prevalence (~5%–25% of ASD) and linguistic descriptions at various ages (Anderson et al., 2007; Bal et al., 2016; Norrelgen et al., 2015). Moreover, we found an even distribution between LI and LU language profiles, in line with reported prevalence rates (Kjelgaard & Tager-Flusberg, 2001; Loucas et al., 2008). The profiles of LU and LI at outcome also matched their classical description, LU’s verbal DQ lying within one standard deviation from TD, while LI’s grammar and vocabulary remained significantly behind (Boucher, 2012). Nonetheless, though our clusters showed important similarities with the three canonical language profiles of ASD, a small subset of autistic preschoolers might occasionally transition between profiles during school-age as shown previously (Pickett et al., 2009). As the Geneva Autism Cohort now follows participants beyond the age of 6 (Bochet, 2022), we plan to track the later trajectories of profiles to estimate their stability over childhood, as well as their association with various comorbidities like Intellectual Disability or Attention Deficit Disorder. Finally, we found no parental stress difference between the language profiles, even though we found that ASD significantly increased parental stress compared to TD (PSI-SF: 90.5 ± 22.8 in ASD versus 59.1 ± 18.0 in TD, see Table 1), which is in line with previous research (Mugno et al., 2007). Consequently, increased familial stress might be a generic characteristic of early ASD diagnosis that is not modulated by the preschooler’s verbal abilities. This result emphasizes the need to monitor and support parental distress in the early years following diagnosis, even when children are facing no/minimal language delay (Fuentes et al., 2021).

Another important contribution of our study is the unprecedented extensive language phenotyping of the early trajectories within ASD language profiles (Chenausky & Tager-Flusberg, 2022). Extensive language phenotyping revealed some patterns of early language acquisition shared by all profiles, and others more specific (see Figure 2). For instance, all language profiles showed a delayed onset of vocabulary acquisition. In TD, the average lexical expansion started at ~1.5 y.o., corresponding to the canonical “*word spurt*” (Nagy & Scott, 2016), whereas this process started after 2 y.o. in LU and LI language profiles (see Figure 2(a)). Regarding word combination, nearly all TD participants mastered two-word phrases by age 2, while most autistic participants did not (only ~25% in LU and < 10% in LI and MV individuals, see Figure 2(d)). Those findings suggest that early language delay could be a common feature of ASD, irrespective of later profile (Hudry et al., 2010). Another interesting finding was that all linguistic domains (vocabulary, grammar, and pragmatics) exhibited similar trajectories within each profile, suggesting that early language delays in ASD undergo no major dissociations between vocabulary and grammar acquisition). We also found some early characteristics that were specific to each profile. For instance, LU’s rates of acquisition were similar to TD once the “*word spurt*” started, resulting in a parallelly shifted trajectory compared to TD (see Figure 2(a) to (c)). In contrast, LI individuals showed slower rates of vocabulary and grammar acquisition compared to both TD and LU. However, some limitations of our extensive language phenotyping method should be stated. Some pragmatic abilities, like implicatures, are acquired later than grammar and vocabulary (Noveck, 2001) and we might have thus missed some subtle nuances between autistic language profiles. Moreover, parent-reported questionnaires have shown less accuracy for individuals with very low verbal abilities, suggesting that the DLPF data in the MV group might be subject to more noise (Eadie et al., 2010). Finally, the DLPF mainly focuses on expressive abilities. Although ASD is globally associated with homogeneous alterations in both expressive and receptive skills (Kjelgaard & Tager-Flusberg, 2001; Kwok et al., 2015; Loucas et al., 2008), we might have missed some subtle expressive/receptive gaps in specific subpopulations (Chen et al., 2024). In conclusion, although our approach is more precise and extensive than verbal aggregate measures, it does not replace a true deep language phenotyping (Chenausky & Tager-Flusberg, 2022). Future studies should combine parent report with direct language assessments, speech sampling and/or implicit measures (e.g. neuroimaging) to provide a more comprehensive and precise language phenotyping, especially for MV participants (Cantiani et al., 2016; Tager-Flusberg et al., 2017).

Finally, we ran some analyses to identify the factors that might have moderated the participants’ profile belonging (see Table 2 and Figure 3) as well as their verbal outcome within their own profile (see Table 3 and Figure 4). In line with previous studies, the MV cluster showed increased proportion of females (Fombonne, 2009; Kirkovski et al., 2013; Nydén et al., 2000) and very low VR abilities at age 2.4 (Anderson et al., 2007). LU and LI individuals differed on almost all non-verbal behavioral moderators at 2.4 y.o. (VR, FM, and SA; see Figure 3). Our results are in line with previous studies pointing toward the role of early developmental difficulties in the emergence of language delays in ASD, for example, early motor (Godel et al., 2023; Hwang & Lee, 2022; LeBarton & Iverson, 2013) and early VR delays (Hellendoorn et al., 2015). Moreover, we found that the participation in an early intervention program (individualized ESDM for 2 years, 15–20 h per week) was moderating the language profile attribution, with higher levels of LU children who received the ESDM compared to MV participants. This result should be explored with a dedicated randomized controlled design to exclude possible inclusion biases. Our results nonetheless provide promising support for the fact that ESDM might have changed the language profile of children who would otherwise have shown a developmental trajectory with the lowest rate of acquisition of language.

**Figure 5.**
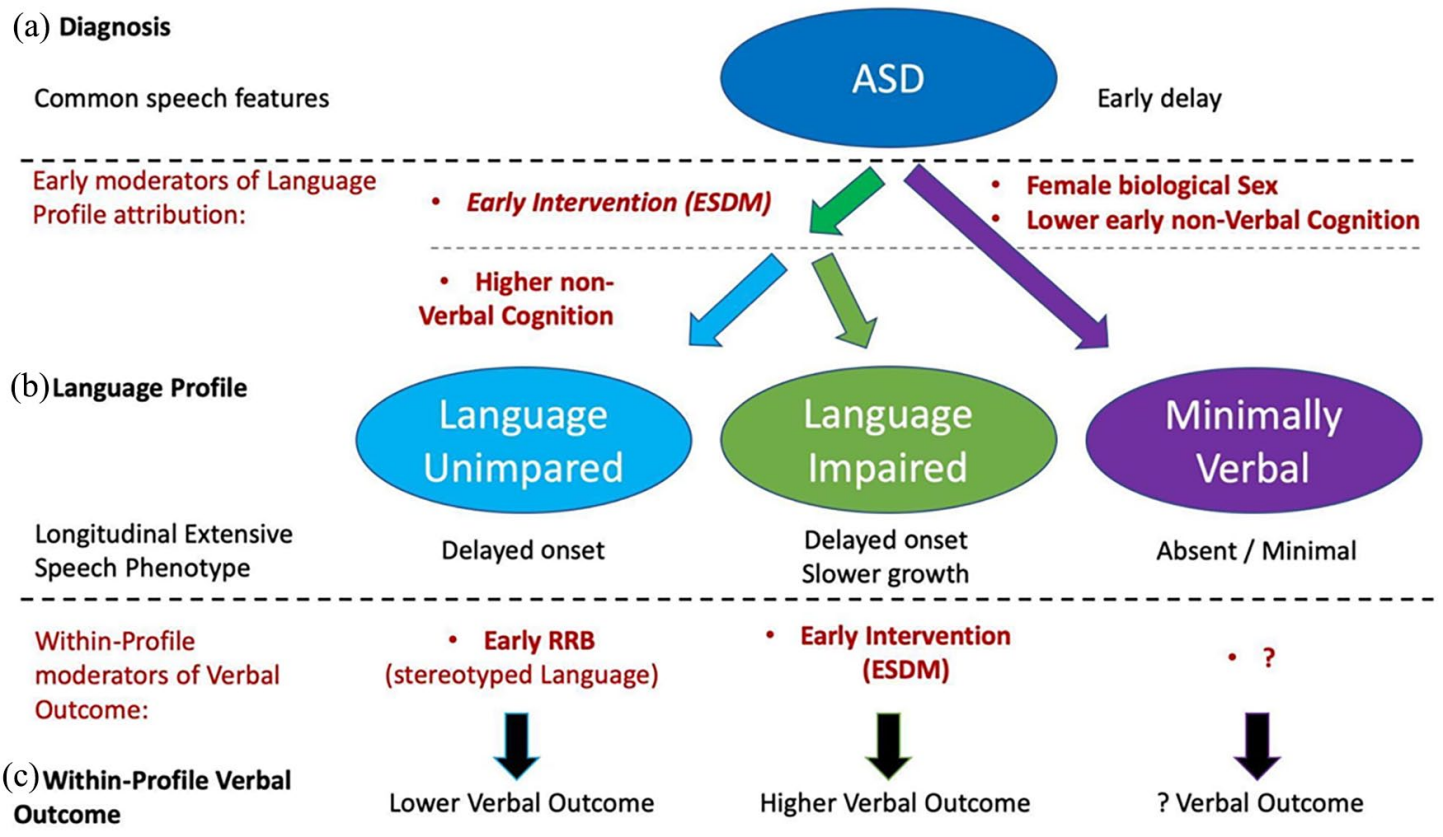
Suggestion of an original theoretical three-layer model for the early emergence of autistic language profiles. Our results suggest that (a) the ASD group presents a common early language characteristic: early delay in all domains. (b) Within ASD, we found three persisting language profiles with their specific moderators (in red) and dynamical extensive language phenotypes. For example, MV profile was specifically associated with female biological sex and very low non-verbal DQ at 2.4 y.o. (c) Then, we found some specific features that explained the within-profile variability in verbal outcome. For example, in LU, early RRB (stereotyped language) were associated with lower verbal outcome. In LI, 2-year early intensive individualized early intervention (Early Start Denver Model, ESDM) was associated with higher verbal outcome. Finally, we did not identify any significant moderator of verbal outcome within MV. DQ: developmental quotient.

Although each ASD language profile exhibited its own specific language trajectories, language difficulties greatly varied within one profile (Kjelgaard & Tager-Flusberg, 2001; Modyanova et al., 2017). Interestingly, plurilingual exposure did not affect verbal outcome. This result has clinical relevance as parents and clinicians often wonder whether to encourage or discourage bilingual exposure in ASD. Our results thus converge with Prévost and Tuller (2022) in the absence of any detrimental effect of early bilingual exposure on autistic language development. In contrast, other early moderators partly explained the within-profile verbal heterogeneity. Higher RRB (stereotyped language) at 2.4 y.o. predicted lower verbal skills 2 years later within LU children (Figure 4(b) and (c)). While early improvement in verbal ability have been shown to lead to later decreased RRB (Paul et al., 2008; Ray-Subramanian & Ellis Weismer, 2012), our results indicated the opposite causality within LU children, that is, lower early RRB associated with higher verbal skills at outcome. Interestingly, we did not find that early SA played any significant role in either profile attribution or within-profile verbal outcome. This result fails to support the existing constructivist models hypothesizing a role of early autistic impairment in social reciprocity on later language acquisition (Chevallier et al., 2012; Kuhl et al., 2005; Tang et al., 2023). Regarding LI individuals, ESDM intervention was the only factor that significantly contributed to moderating individuals’ verbal outcome (see Figure 4(a)). While important language improvement after ESDM has been one of the most consistent findings (Fuller et al., 2020), no study to date has explored ESDM as a moderator of verbal outcome within stratified language profiles. Our study suggests that the well-established ESDM effect on language might be mostly driven by the LI profile. Considering the lack of any ESDM significant effect on verbal outcome within the LU profile, we hypothesize that the verbal outcome of LU children might have reached a ceiling potential as their outcome values were very close to TD norms (see Figure 1) with near-TD rates of language acquisition (see Figure 2). The outcome verbal values being so close to the optimal value (100) might only leave a small lever to ESDM to affect language outcome. However, ESDM might have improved other domains that we did not test (e.g. social skills or autonomy) or subtle verbal skills that were not assessed by the outcome verbal DQs. When it comes to MV individuals, many factors might explain why ESDM did not reach significance threshold as a moderator of verbal outcome in this specific subgroup. First, ESDM might have affected the MV cluster attribution (e.g. switching a participant’s MV trajectory to a LI one) rather than moderating the verbal outcome within this profile (see Table 2). Alternatively, some methodological issues might explain why we failed to find any significant moderators of language outcome within the MV profile. First, MV individuals displayed the lowest statistical power due to its smaller sample, negative results should thus be taken with more caution given the higher probability of false negatives. Yet, MV individuals showed important heterogeneity in their own extensive language phenotype (e.g. 17.2% of MV mastered two-word phrase production), raising the question of which moderator might have impacted this heterogeneity. Moreover, Tager-Flusberg et al. (2017) have argued that behavioral evaluations and parent-report might give a highly noisy impression of the true MV participants’ language abilities. Consequently, future studies with larger MV sample size and/or using implicit measures of language abilities are warranted to understand the causes of this within-MV heterogeneity (Cantiani et al., 2016; Tager-Flusberg et al., 2017). To summarize and integrate our main findings, we suggested a three-layer model that is presented on Figure 5. In this model, some language features are shared by all autistic language profiles, whereas others are specific to one profile. Moreover, some features moderate the participants’ profile attribution, while others moderate their verbal outcome within their own profile.

## Conclusion

In conclusion, our data-driven approach successfully identified the three canonical language profiles of ASD by the age of 4.4. Moreover, we described each profile’s language trajectory, and identified some moderators of verbal outcome. First, early language delay was shared across the whole autistic spectrum, and there were no dissociations between linguistic domains (vocabulary, grammar, and pragmatics) in any of the three language profiles (see Figure 5(a)). Then, each profile had its own linguistic dynamic patterns (see Figure 5(b)), for example, LU children showed a shifted trajectory compared to TD (pure delay), while LI children underwent a slower slope of acquisition (delay and slowdown). Some factors moderated the participants’ profile attribution, for example, female biological sex and early non-verbal developmental delays were associated with a MV profile attribution. Moreover, certain factors specifically moderated the verbal outcomes within one profile, while sparing the others (see Figure 5(c)). For instance, early stereotyped language was associated with later verbal skills in LU individuals. Disentangling the hierarchy of early factors involved in autistic language difficulties has major relevance in the field of personalized medicine (Ozomaro et al., 2013). Here, for instance, we found that early ESDM intervention may have played a role in the profile attribution and that its efficacy on language outcome might have been especially important within the LI profile. Such results are crucial to inspire more targeted intervention models and guidelines. Overall, our study emphasizes the application of longitudinal extensive language phenotyping on autistic preschoolers to better understand the emergence of ASD language profiles (Chenausky & Tager-Flusberg, 2022).

## Supplemental Material

sj-docx-1-aut-10.1177_13623613241253015 – Supplemental material for Early trajectories and moderators of autistic language profiles: A longitudinal study in preschoolers Supplemental material, sj-docx-1-aut-10.1177_13623613241253015 for Early trajectories and moderators of autistic language profiles: A longitudinal study in preschoolers by Kenza Latrèche, Michel Godel, Martina Franchini, Fiona Journal, Nada Kojovic and Marie Schaer in Autism
