# Supplementary material for: Early trajectories and moderators of autistic language profiles: A longitudinal study in preschoolers
Source: Autism. 2024 May 21;28(12):3043–62. doi: 10.1177/13623613241253015 (PMC11575100; doi:10.1177/13623613241253015)

1. **B.**


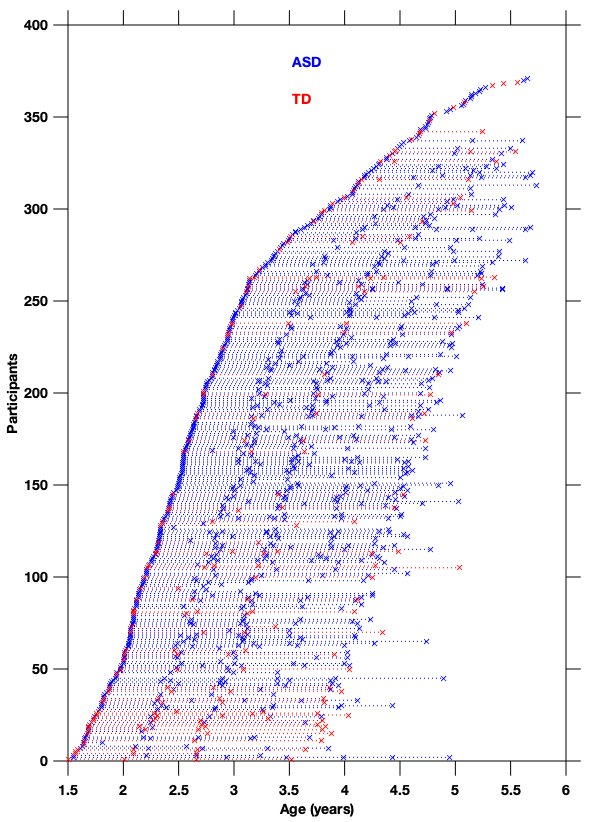

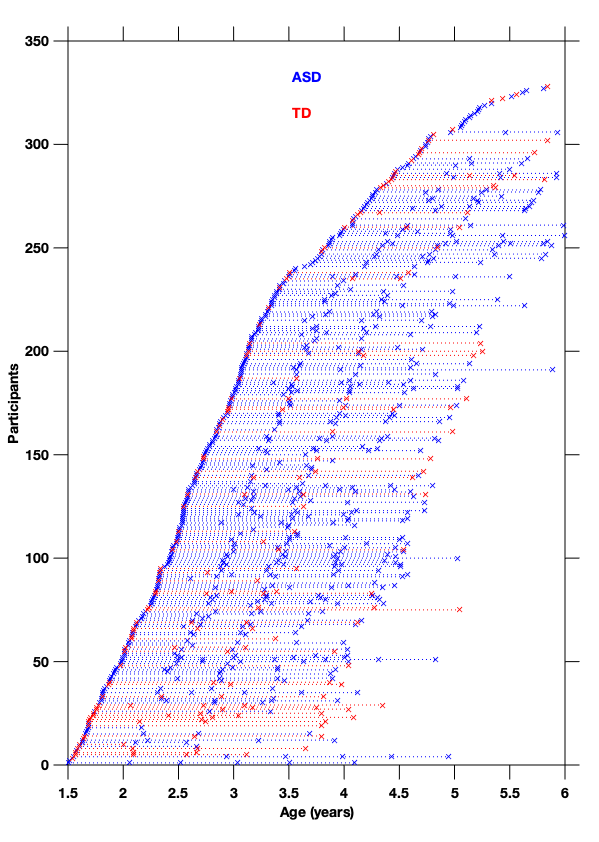


**Fig S1. Illustration of longitudinal recruitment of our sample.** (**A**) Total sample. (**B**) Sub-sample of participants with a DLPF.

A)


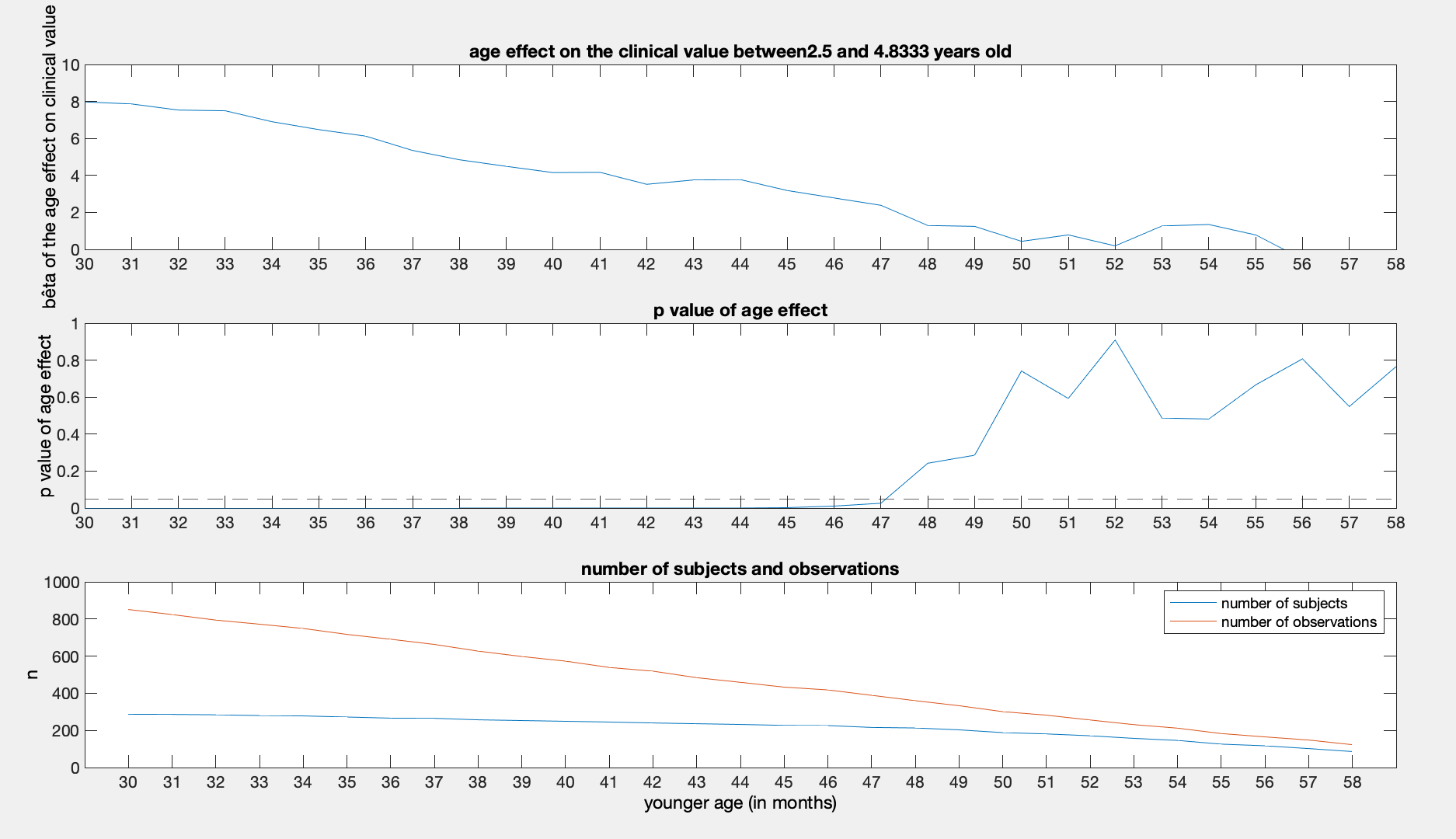


B)


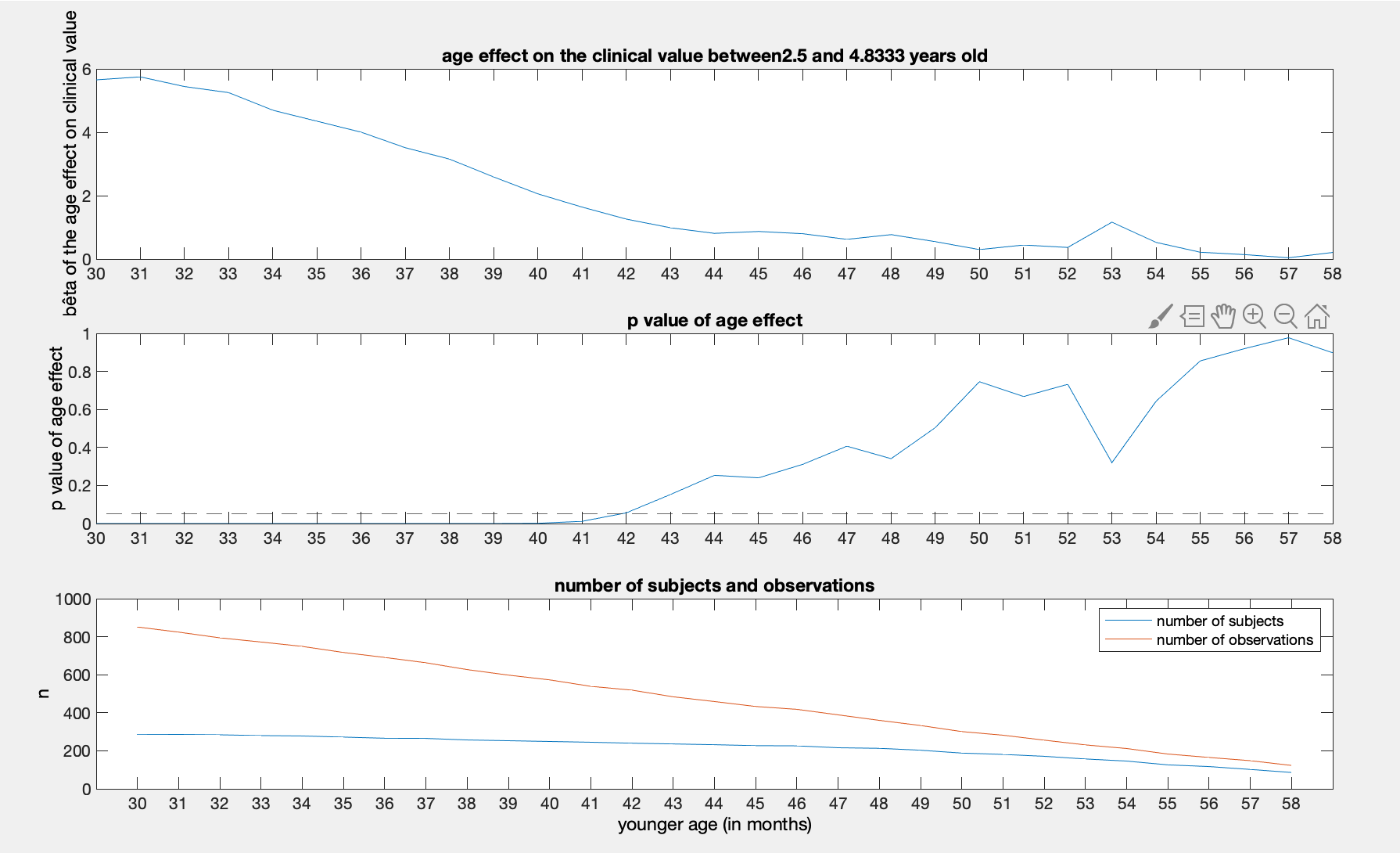


**Fig S2. Best age for clustering using a 12-month sliding window within ASD group.** (**A**) Receptive language DQ (**B**) Expressive language DQ


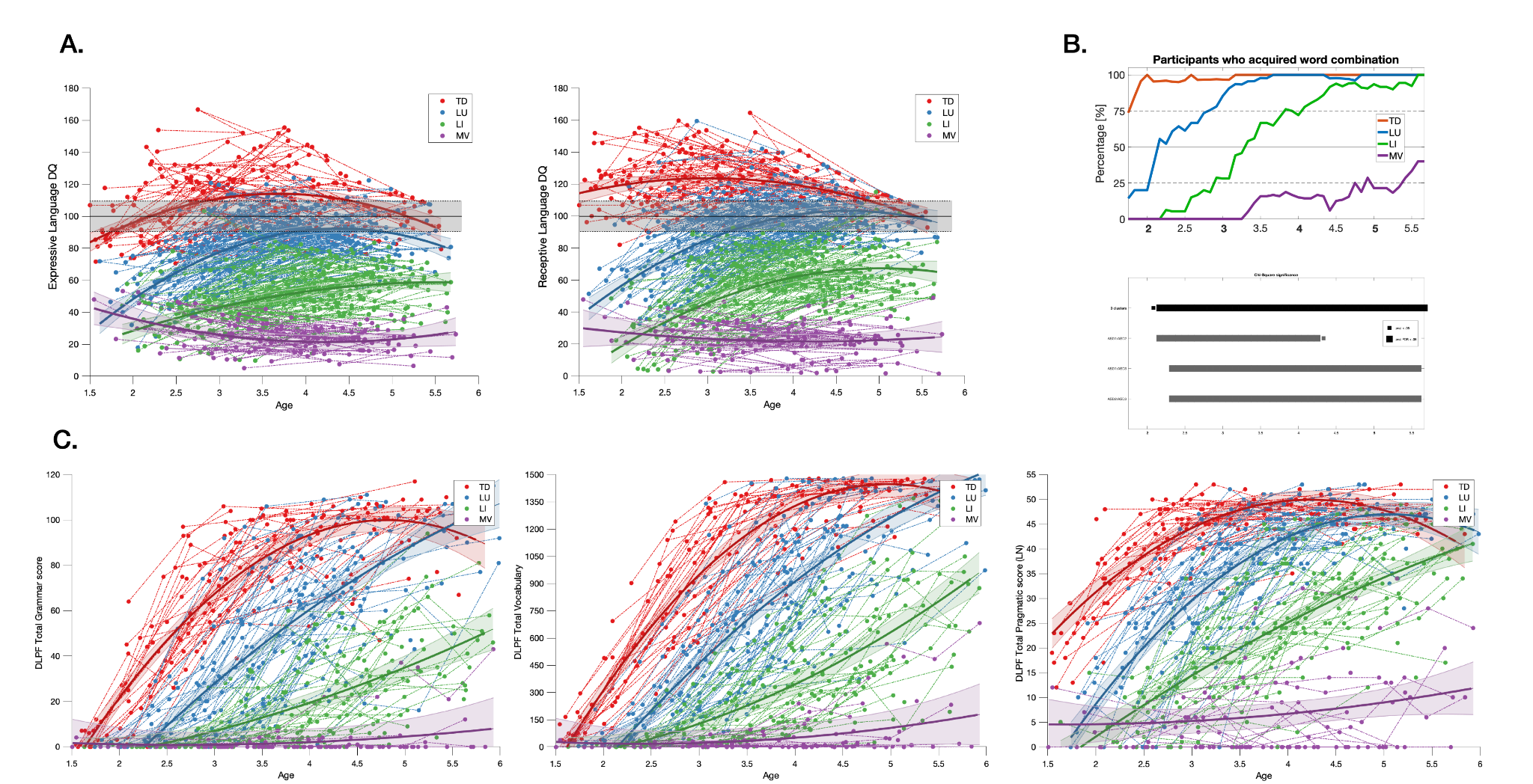


**Fig S3. Longitudinal speech phenotypes in the three ASD language profiles.** (**A**) Expressive and Receptive Language DQs. (**B**) DLPF Proportion of word combination. (**C**) DLPF Vocabulary, Grammar, and Pragmatic scores. TD (in red) were not included in the statistical comparison and their trajectory is only displayed for illustration purposes. The colored bands around the estimated group-level trajectory indicate the 95% confidence interval. DLPF: Questionnaire du développement du langage productif en français ; DQ: Developmental Quotient ; LI: Language Impaired ; LU: Language Unimpaired ; MV: Minimally Verbal ; TD: Typical Development.

**
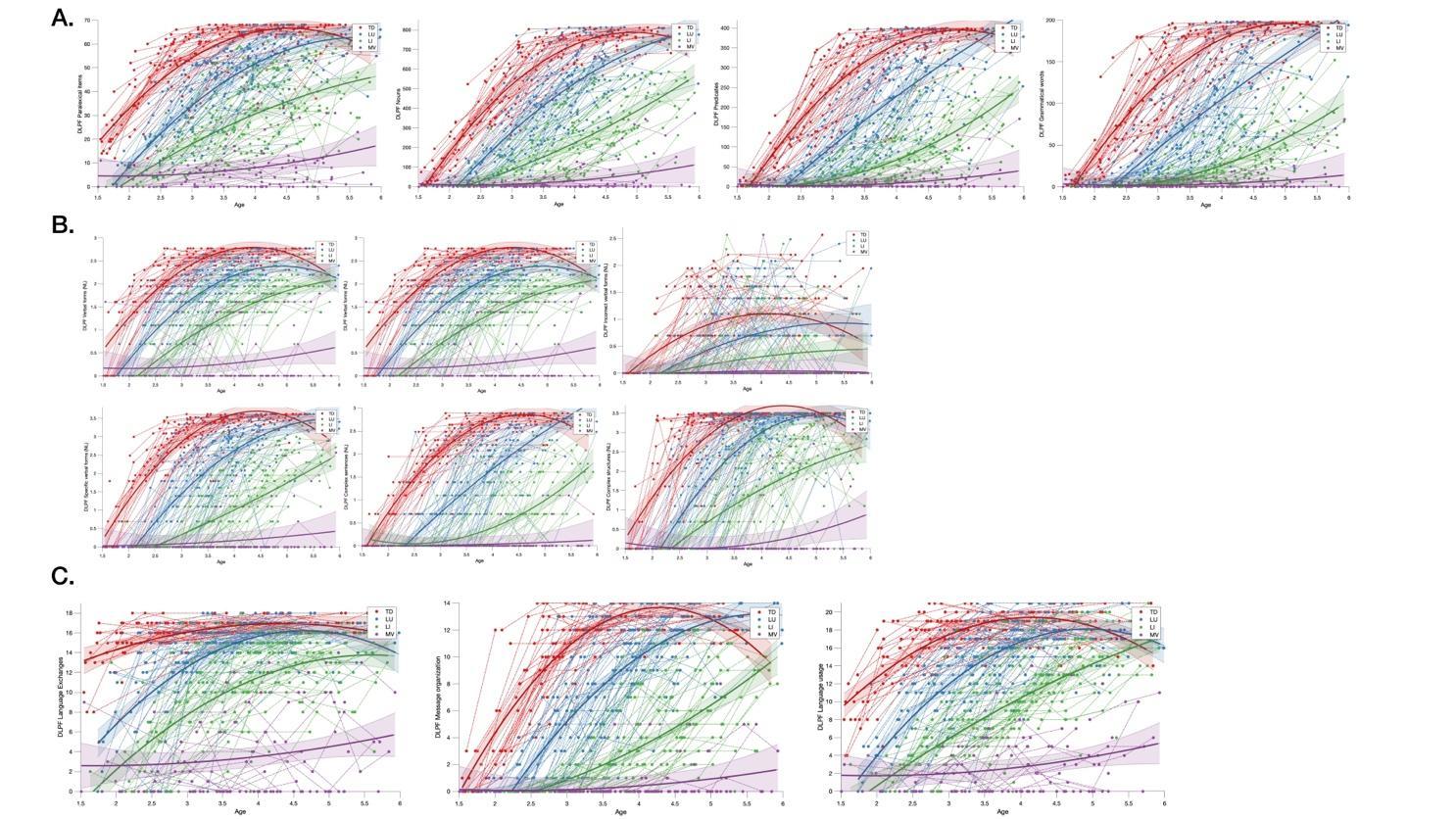
**

**Fig S4. Longitudinal speech phenotypes in three autism language profiles measured with DLPF subscales** in (**A**) Vocabulary (**B**) Grammar (**C**) Pragmatics. TD (in red) were not included in the statistical comparison and their trajectory is only displayed for illustration purposes. The colored bands around the estimated group-level trajectory indicate the 95% confidence interval. DQ: Developmental Quotient ; DLPF: *Questionnaire du développement du langage productif en français* ; TD: Typical Development ; MV: Minimally Verbal ; LI: Language Impaired ; LU: Language Unimpaired.

**Table S1.** Behavioral characteristics of the three ASD clusters at outcome.

At the age of 4.4, our three clusters displayed the typical features of the three canonical ASD phenotypes described in the literature: Minimally Verbal (MV), Language Impaired (LI) and Language Unimpaired (LU) (Boucher 2012; Schaeffer et al. 2023; Tager-Flusberg 2006). Results of ANOVA across three clusters are displayed, with post-hoc head-to-head comparisons.

| **Cluster Characteristics** | **LU** | **LI** | **MV** | **P-Val** | **LU/LI** | **LU/MV** | **LI/MV** |
| --- | --- | --- | --- | --- | --- | --- | --- |
| **Description at Outcome** |  |  |  |  |  |  |  |
| Number of participants | 86 | 85 | 44 |  |  |  |  |
| Age | 4.4 (0.3) | 4.4 (0.3) | 4.5 (0.4) | .331 |  |  |  |
| ADOS CSS total | 6.8 (1.7) [n=64] | 7.2 (1.8) [n=67] | 8.6 (1.3) [n=33] | **< .001 (KW)** | .666 (MW) | **< .001 (MW)** | **< .001 (MW)** |
| ADOS CSS SA | 5.7 (1.8) | 6.2 (1.8) | 7.6 (1.6) | **< .001 (KW)** | .564 (MW) | **< .001 (MW)** | **< .001 (MW)** |
| ADOS CSS RRB | 8.5 (1.6) | 8.9 (1.7) | 9.7 (0.8) | **< .001 (KW)** | .123 (MW) | **< .001 (MW)** | .114 (MW) |
| Composite DQ | 99.9 (11.0) | 67.9 (11.3) | 33.7 (8.6) | **< .001** | **< .001** | **< .001** | **< .001** |
| Expressive Language DQ | 90.0 (11.5) | 55.2 (13.8) | 21.6 (6.9) | **< .001** | **< .001** | **< .001** | **< .001** |
| Receptive Language DQ | 101.5 (14.1) | 62.1 (14.3) | 21.7 (10.6) | **< 0.001** | **< .001** | **< .001** | **< .001** |
| Visual Reception DQ | 109.5 (16.7) | 80.6 (17.2) | 44.7 (12.3) | **< .001** | **< .001** | **< .001** | **< .001** |
| Fine Motor DQ | 98.5 (16.3) | 73.8 (15.2) | 46.7 (12.2) | **< .001** | **< .001** | **< .001** | **< .001** |
| Vocabulary | 1091 (289) [n=62] | 406 (249) [n=52] | 103 (156) [n=27] | **< .001** | **< .001** | **< .001** | **< .001** |
| Grammar | 55.5 (30.0) [n=61] | 23.9 (17.4) [n=49] | 4.0 (10.2) [n=27] | **< .001** | **< .001** | **< .001** | **< .001** |
| Word Combination | 61 (98.4%) [n=62] | 44 (84.6%) [n=52] | 5 (17.2%) [n=29] | **< .001** | **.021** | **< .001** | **< .001** |
| Pragmatic | 44.7 (28.6) [n=59] | 28.6 (10.2) [n=47] | 8.5 (9.5) [n=29] | **< .001** | **< .001** | **< .001** | **< .001** |
| PSI Stress Total | 87.2 (26.6) [n=53] | 91.4 (30.6) [n=48] | 101.1 (19.3) [n=26] | .104 |  |  |  |

**Table S2.** Results of the mixed model analysis comparing developmental and linguistic skills between TD and the three ASD clusters.

Note: *P-*value group effect evaluates the difference between mean linguistic scores in the two groups whereas *p*-value interaction refers to the difference in the shape of the linguistic trajectory. The significance threshold was fixed at 0.05. All the p-values are Bonferroni corrected. LU: Language Unimpaired; LI: Language Impaired; MV: Minimally Verbal

|  | LU *vs.* LI *vs.* MV | |  | LU *vs.* LI | |  | LI *vs.* MV | |  | LU *vs.* MV | |
| --- | --- | --- | --- | --- | --- | --- | --- | --- | --- | --- | --- |
|  | *p*-val group effect | *p-*val interaction |  | *p*-val group effect | *p-*val interaction |  | *p*-val group effect | *p-*val interaction |  | *p*-val group effect | *p-*val interaction |
| MSEL Total DQ | <0.001 | <0.001 |  | <0.001 | <0.001 |  | <0.001 | <0.001 |  | <0.001 | <0.001 |
| MSEL VR DQ | <0.001 | <0.001 |  | <0.001 | 0.034 |  | <0.001 | <0.001 |  | <0.001 | <0.001 |
| MSEL FM DQ | <0.001 | <0.001 |  | <0.001 | <0.001 |  | <0.001 | <0.001 |  | <0.001 | <0.001 |
| MSEL RL DQ | <0.001 | <0.001 |  | <0.001 | <0.001 |  | <0.001 | <0.001 |  | <0.001 | <0.001 |
| MSEL EL DQ | <0.001 | <0.001 |  | <0.001 | <0.001 |  | <0.001 | <0.001 |  | <0.001 | <0.001 |
| DLPF Total Vocabulary Score | <0.001 | <0.001 |  | <0.001 | <0.001 |  | <0.001 | <0.001 |  | <0.001 | <0.001 |
| DLPF Total  Grammar Score | <0.001 | <0.001 |  | <0.001 | <0.001 |  | <0.001 | <0.001 |  | <0.001 | <0.001 |
| DLPF Total  Pragmatic Score | <0.001 | <0.001 |  | <0.001 | <0.001 |  | <0.001 | <0.001 |  | <0.001 | <0.001 |

**Supplementary Material 1. Results for the 15 models tested by the TwoStep Cluster Analysis**

AIC, AIC change (from previous model), ratio of AIC changes (using the 2-cluster model value as reference) and ratio of Distance Measures (using log-likelihood method) for each model tested. Please note that the Ratio of AIC changes of the 2-cluster solution is always 1.000, by definition.

Plots of the Ratio of AIC changes and the Ratio of Distance Measures. The optimal cluster solution was automatically determined by SPSS® based on a compromise between the highest ratio of AIC changes and the highest ratio of Distance Measures. Here, the 3-cluster solution combines a high value in both measures.


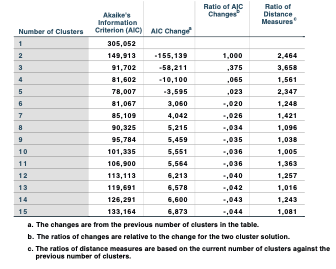


Plots of the Ratio of AIC changes and the Ratio of Distance Measures. The optimal cluster solution was automatically determined by SPSS® based on a compromise between the highest ratio of AIC changes and the highest ratio of Distance Measures. Here, the 3-cluster solution combines a high value in both measures.


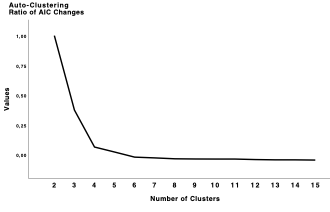

Supplement: sj-docx-1-aut-10.1177_13623613241253015 – Supplemental material for Early trajectories and moderators of autistic language profiles: A longitudinal study in preschoolers [file sj-docx-1-aut-10.1177_13623613241253015.docx]
